# Stress and human health in diabetes: A report from the 19^th^ Chicago Biomedical Consortium symposium

**DOI:** 10.1017/cts.2023.646

**Published:** 2023-11-20

**Authors:** Raghavendra G. Mirmira, Rohit N. Kulkarni, Pingwen Xu, Tina Drossos, Krista Varady, Kristen L. Knutson, Sirimon Reutrakul, Pamela Martyn-Nemeth, Robert M. Sargis, Amisha Wallia, Arleen M. Tuchman, Jill Weissberg-Benchell, Kirstie K. Danielson, Scott A. Oakes, Celeste C. Thomas, Brian T. Layden, Sarah C. May, Michelle Burbea Hoffmann, Eleonora Gatta, Julian Solway, Louis H. Philipson

**Affiliations:** 1 Department of Medicine, Kovler Diabetes Center, The University of Chicago, Chicago, IL, USA; 2 Department of Medicine, Islet Cell and Regenerative Biology, Joslin Diabetes Center, Beth Israel Deaconess Medical Center, Harvard Stem Cell Institute, Boston, MA, USA; 3 Division of Endocrinology, Diabetes and Metabolism, Department of Medicine, University of Illinois Chicago, Chicago, IL, USA; 4 Department of Psychiatry and Behavioral Neuroscience, Pritzker School of Medicine, The University of Chicago, Chicago, IL, USA; 5 Department of Kinesiology and Nutrition, University of Illinois Chicago, Chicago, IL, USA; 6 Department of Neurology, Center for Circadian and Sleep Medicine, Northwestern University Feinberg School of Medicine, Chicago, IL, USA; 7 Department of Biobehavioral Nursing Science, University of Illinois Chicago College of Nursing, Chicago, IL, USA; 8 Department of Medicine, Section of Endocrinology, Diabetes and Metabolism, Jesse Brown VA Medical Center, Chicago, IL, USA; 9 Department of Medicine, Division of Endocrinology, Metabolism, and Molecular Medicine, Feinberg School of Medicine, Northwestern University, Chicago, IL, USA; 10 Department of History, Vanderbilt University, Nashville, TN, USA; 11 Department of Psychiatry and Behavioral Sciences, Ann & Robert H. Lurie Children’s Hospital of Chicago, Feinberg School of Medicine, Northwestern University, Chicago, IL, USA; 12 Department of Pathology, The University of Chicago, Chicago, IL, USA; 13 Section of Adult and Pediatric Endocrinology, Diabetes and Metabolism, The University of Chicago, Chicago, IL, USA; 14 Chicago Biomedical Consortium, Evanston, IL, USA; 15 Department of Medicine, University of Chicago, Chicago, IL, USA; 16 Department of Medicine and Pediatrics, Section of Adult and Pediatric Endocrinology, Diabetes and Metabolism, The University of Chicago, Chicago, IL, USA

**Keywords:** Diabetes, stress, beta cells, metabolic syndrome, diabetes technology, healthcare, community

## Abstract

Stress and diabetes coexist in a vicious cycle. Different types of stress lead to diabetes, while diabetes itself is a major life stressor. This was the focus of the Chicago Biomedical Consortium’s 19^th^ annual symposium, “Stress and Human Health: Diabetes,” in November 2022. There, researchers primarily from the Chicago area met to explore how different sources of stress – from the cells to the community – impact diabetes outcomes. Presenters discussed the consequences of stress arising from mutant proteins, obesity, sleep disturbances, environmental pollutants, COVID-19, and racial and socioeconomic disparities. This symposium showcased the latest diabetes research and highlighted promising new treatment approaches for mitigating stress in diabetes.

## Introduction

Stress – both mental and physical – has a negative impact on our overall health. The biological wear and tear that occurs as the body adjusts to chronic stress inflicts widespread damage across the metabolic, cardiovascular, and immune systems [1,2]. These stress-induced damages may trigger the onset of disease or worsen preexisting health conditions. In diabetes, patients experience high levels of mental and physiological stress. Although diabetes prevalence continues to rise globally [3], we are just beginning to realize the consequences of stress on diabetes progression.

Diabetes is a metabolic disease characterized by high blood glucose levels. Over time, elevated blood glucose levels damage nerves, blood vessels, and organs, resulting in various health complications that may be life-threatening. The most common forms of diabetes result from the body’s inability to produce insulin in pancreatic beta cells (type 1 diabetes) or to respond to insulin (type 2 diabetes). Less common forms include gestational diabetes (where hormones block the efficient use of insulin) and monogenic diabetes. Whereas monogenic diabetes is caused by mutation of a single gene, common forms of diabetes are typically caused by multiple factors, including genetic and environmental factors. Moreover, diabetes can involve dysfunction of the pancreas itself or other insulin-responsive tissues (e.g., liver, muscle, and/or fat). As chronic stress affects the whole body, there are many weak points where stress can trigger and/or exacerbate diabetes progression.

**Figure 1.**
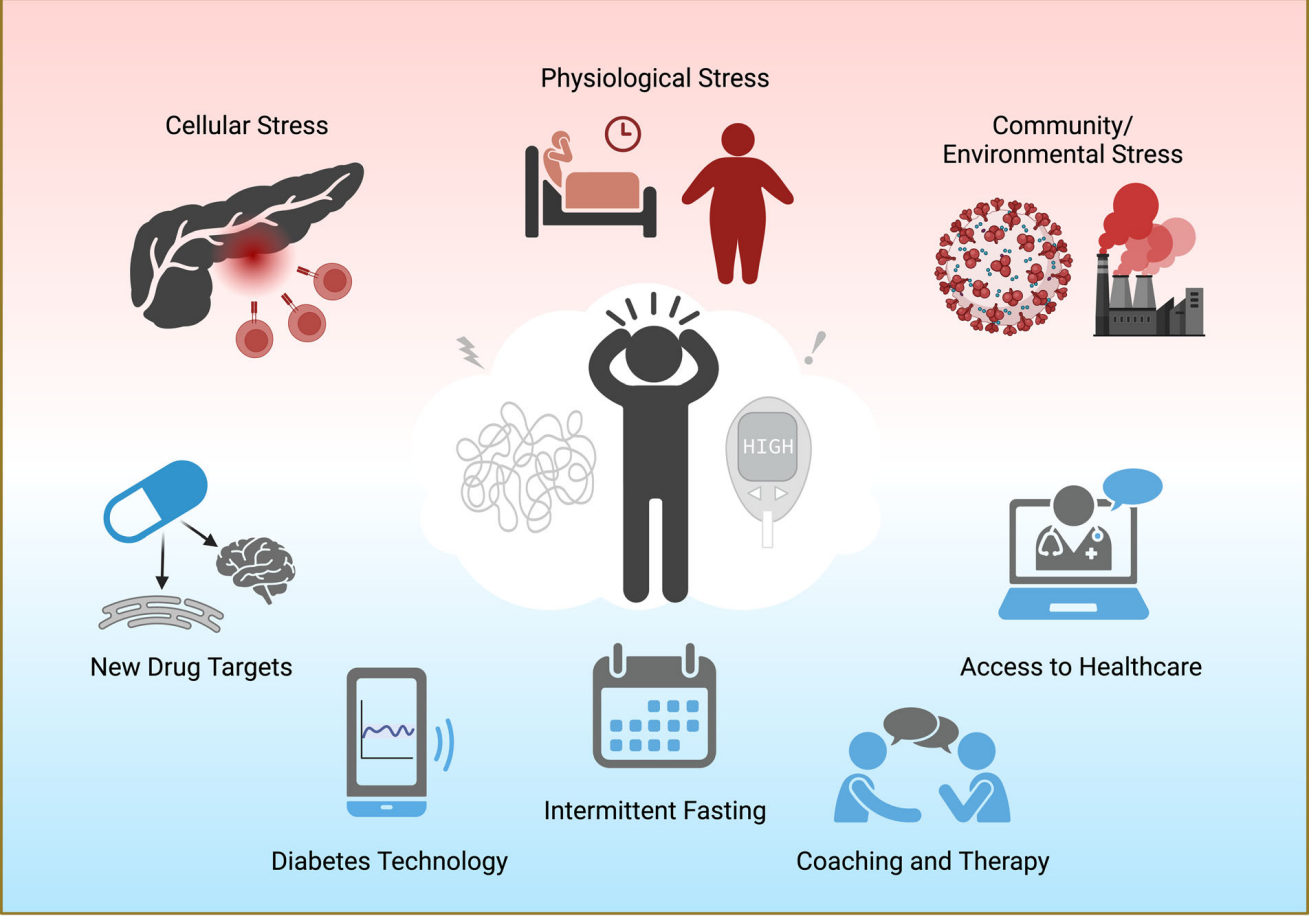
The multifaceted forms of stress in diabetes. Cellular inflammation, poor sleep health, obesity, COVID-19 stress, and exposure to diabetogenic pollutants lead to worsening health outcomes and emotional distress in patients with diabetes. Possible ways to mitigate stress and diabetes include drugs targeting new cellular pathways (such as ER stress and brain estrogen signaling), continuous glucose monitors, intermittent fasting, cognitive behavioral therapy, and better access to quality healthcare, particularly for groups with health disparities. Created with BioRender.com.

Stress in diabetes can take many forms (Fig. 1). At the cellular level, oxidative and endoplasmic reticulum (ER) stress induce pancreatic beta cell dysfunction and death, resulting in the loss of insulin secretion [4]. Physiological stressors, like obesity and sleep disturbances, make it more difficult to initiate lifestyle changes or cope with day-to-day diabetes management. Community-wide stressors, such as environmental pollutants and the COVID-19 pandemic, have a disproportionate effect on racial/ethnic minorities and low socioeconomic households and may prevent access to healthcare or diabetes technologies [5]. The goal of the 19^th^ Chicago Biomedical Consortium symposium “Stress and Human Health: Diabetes” held November 2022 was to explore the various forms of stress in diabetes and discuss how new treatment approaches may improve both stress and diabetes outcomes.

### Stress at the Cellular Level

In diabetes, pancreatic beta cells are at the epicenter of cellular stress. Beta cells reside within the endocrine (hormone-secreting) tissue of the pancreas and secrete insulin to lower blood glucose levels. During type 1 diabetes, beta cells are damaged or destroyed by an aggressive T-cell-mediated attack and related inflammation. Recent research has led to a critical breakthrough – that, during inflammatory stress, beta cells produce aberrant proteins that serve as neoantigens to initiate or exacerbate autoimmunity [6–8]. This was the focus of Raghavendra Mirmira’s talk entitled “New Perspectives on Type 1 Diabetes: The Beta Cell Under Stress,” in which he discussed how therapeutically targeting stress pathways in beta cells might provide a new approach to treating type 1 diabetes. One pro-inflammatory pathway that could be targeted in beta cells involves 12/15-lipoxygenase, an enzyme that converts arachidonic acid into a precursor of the inflammatory lipid 12-hydroxyeicosatetraenoic acid [9] Research from Dr Mirmira’s laboratory has shown that deletion of 12/15-lipoxygenase protects against type 1 diabetes in mice by enhancing immune suppression *via* programmed death ligand 1 [10]. He also showed that the polyamine biosynthesis pathway leads to ER stress and inflammation in beta cells [11]. Both preclinical [12] and in-progress clinical trials show promise in treating type 1 diabetes with the polyamine biosynthesis inhibitor difluoromethylornithine. While most clinical trials to date have focused on targeting the adaptive immune system (with limited success) [13–16], by focusing more on the beta cells, we may find new approaches that complement existing immune-based approaches to treating T1D.

The exocrine (digestive enzyme-secreting) tissue of the pancreas may trigger beta cell stress. In his keynote presentation “Diabetes – A Consequence of Miscommunication Between Exocrine and Endocrine Cells?,” Rohit N. Kulkarni addressed two specific failures in exocrine-endocrine crosstalk that could promote diabetes pathogenesis. The first example is maturity onset diabetes of the young type 8 (MODY8), a type of monogenic diabetes, in which individuals with a mutation in the carboxy ester lipase (CEL) gene, expressed in the non-endocrine part of the pancreas, develop diabetes in adulthood [17]. Dr Kulkarni showed that acinar cells of the exocrine pancreas secrete the mutant CEL protein, which is likely taken up by beta cells where it is unable to be degraded, resulting in ER stress, compromised insulin secretion, and beta cell death [17]. Further studies are necessary to determine exactly how the CEL protein is transferred from acinar cells to beta cells and whether this involves extracellular vesicles and/or exosomes. Second, Dr Kulkarni showed that acinar cells secrete pancreatic elastase (PE), which inactivates a cell surface growth receptor on beta cells called protease-activated receptor-2, triggering a signaling cascade that leads to beta cell death [18]. In humans, when PE is overactive due to a loss-of-function mutation in its inhibitor SERPINB1, diabetes risk increases [19,20]. Similarly, a missense mutation in pancreatic elastase 3B that increases PE expression leads to an inherited syndrome of pancreatitis, diabetes, and pancreatic ductal adenocarcinoma [21]. These examples show clear crosstalk between the exocrine and endocrine pancreas, which were previously thought to be two separate entities, and illustrate the importance of the cellular microenvironment in influencing beta cell stress in diabetes.

### Stress at the Physiological Level

### Stress and Obesity

Metabolic syndrome is a cluster of conditions – including obesity, high fasting blood glucose, high blood pressure, and dyslipidemia – that together put tremendous stress on the body and greatly increase the risk of developing type 2 diabetes, obesity, cardiovascular disease, and other health problems [22]. However, recent research has uncovered a protective role of estrogen hormone during the pathogenesis of metabolic syndrome in both men and women [23]. Pingwen Xu discussed the protective role of estrogen in his talk entitled “Estrogens in the Brain and Metabolic Adaptation to Temperature and Nutritional Stresses.” The protective effects of estrogen are mostly mediated through estrogen receptor α (ERα), and mutations in ERα can lead to obesity in humans [24–26]. Several brain regions express high levels of ERα and mediate specific metabolic functions of estrogens, such as reducing food intake [27], inhibiting binge-like eating [28], stimulating brown adipose tissue (BAT) thermogenesis to increase energy expenditure [27,29], and promoting locomotion to elevate energy expenditure [30]. More recently, neural circuits have been identified that respond to temperature and nutritional stresses and mediate different effects of estrogens on metabolic adaptation. Dr Xu and colleagues identified a subpopulation of ERα neurons – in the ventrolateral region of the ventromedial hypothalamus and projecting to the downstream dorsal raphe nuclei – that integrate ambient temperature and nutritional/metabolic states of animals to engage adaptive changes in physical activity and BAT thermogenesis [31]. Additionally, ERα neurons in the medial preoptic area of the hypothalamus coordinate energy deficiency-induced hypothermic and hypometabolic responses, known as torpor, in female mice [32]. The neural mechanisms by which estrogen/ERα regulates metabolism are complex. By deciphering how estrogens interact with specific neural circuits in response to various stresses, we may identify new targets for treating metabolic syndrome and obesity.

Individuals with obesity are up to 80 times more likely to develop type 2 diabetes than those with a body mass index < 22 kg/m^2^ [33]. Obesity serves not only as a risk factor for type 2 diabetes but also as a source of added stress in diabetes management. In “Nutrition and Obesity: Key Factors in Diabetes Management,” Tina Drossos highlighted some of the challenges in treating patients with obesity and diabetes. People with obesity may struggle with a multitude of obstacles that impede weight loss. These obstacles include pain from physical activity, lack of social support, psychological distress, unhealthy coping strategies (such as overeating), and lack of access to affordable, nutritious food. Further, these individuals are at increased rates of medical complications and mental health disorders [34,35]. In order to treat this complex condition, The American Association of Diabetes Educators promotes a self-care behavior framework in seven steps: physical activity, healthy eating, problem-solving, adherence to medications, monitoring, being active, and reducing risks [36]. As these steps can be extremely challenging for individuals with comorbid obesity, it is important for providers to continuously assess and refer patients who are struggling with diabetes management. For mental health issues, such as diabetes distress and diabetes burnout, self-reporting can be effective. However, a structured or semi-structured clinical interview may be used to better assess symptoms, adherence, and motivation. The top mental health interventions include cognitive behavioral therapy (CBT) for obesity and CBT for adherence and depression [37,38], acceptance and commitment therapy, and mindfulness [39]. Along those lines, Dr Drossos and colleagues showed that emotion-focused behavioral interventions are particularly effective, as they both reduce hemoglobin A1c (HbA1c) and alleviate symptoms of diabetes distress [40]. In sum, well-planned assessments and interventions will be critical in helping patients with comorbid obesity and diabetes manage their complex diseases.

A weight loss intervention that has gained popularity in recent years is intermittent fasting. In this diet regimen, periods of eating are alternated with periods of fasting – without restricting certain food groups or counting calories. Thus, diet tolerability is increased. Two types of intermittent fasting are time-restricted eating and alternate-day fasting. In time-restricted eating, the eating window is confined to a set number of hours per day (usually 4–8 hours). In alternate-day fasting, individuals alternate a fast day (where 0-500 kcal are consumed) with a feast day (where one is permitted to eat ad libitum). In her talk entitled “Health Benefits of Intermittent Fasting,” Krista Varady detailed the metabolic changes that may result from these diets. In human trials, both types of intermittent fasting produced mild to moderate weight loss (3%–7%) and reduced energy intake (10-30% from baseline) [41]. In work performed by Dr Varady and others, alternate-day fasting lowered both systolic and diastolic blood pressure by approximately 5%–10% in individuals with baseline hypertension [41–46]. Time-restricted eating also modestly lowered blood pressure – systolic (by 4%–9%) and diastolic (by 7%–9%) – in some but not all studies [41]. Alternate-day fasting led to 10%–23% reductions in LDL cholesterol [47–49], whereas time-restricted eating had no effect [50–56]. Although fasting blood glucose levels were not altered, both intermittent fasting regimens showed promise in lowering fasting insulin and insulin resistance in healthy and prediabetic individuals with obesity [41]. In human trials of time-restricted eating, Dr Varady’s group and others have observed potent reductions in oxidative stress markers, such as 8-isoprostane [51,55]. Only mild and temporary adverse effects (e.g., nausea, dizziness, and headaches) have been associated with the diets. Thus, intermittent fasting is a safe way to improve cardiometabolic health by lowering blood pressure, insulin resistance, and oxidative stress, but intermittent fasting’s ability to improve plasma lipids and markers of inflammation remains uncertain.

### Stress and Sleep Disturbances

Obesity and diabetes are associated with poor sleep health – shorter sleep duration, poor sleep quality, later sleep timing, and reduced feelings of restoration and alertness [57]. Sleep disturbances can impact glucose metabolism, as Kristen Knutson discussed in “Sleep Health and Metabolic Disorders: A Review of the Evidence.” In sleep deprivation studies, people who slept for 4 hours/night for 6 nights, 5 hours/night for 7 nights, or 5.5 hours/night for 14 nights had reduced glucose tolerance, insulin sensitivity, and disposition index [58]. Administering sound periodically throughout the night to lower sleep quality also impaired glucose metabolism [58]. Likewise, shifting sleep schedule to the daytime (mimicking shiftwork) impaired glucose tolerance, reduced energy expenditure, and lowered leptin levels [59]. To understand the long-term effects of sleep disturbances, Dr Knutson says we must also examine sleep outside the laboratory in population-based studies. So far, several observational studies have reported associations between habitual sleep patterns and risk of metabolic disorders. Specifically, short sleep and poor quality sleep have both been associated with risk of incident diabetes and incident obesity [58]. Poor sleep health among patients with diabetes is also related to disease management as poor sleep and sleep disorders have been linked to higher HbA1c and several diabetic complications, such as retinopathy, nephropathy, neuropathy, and macrovascular complications [60]. Poor sleep health is also associated with depression, lower quality of life, and mortality among those with diabetes [61,62]. Fortunately, sleep health is modifiable and therefore could be a favorable new strategy, or adjuvant therapy, to improve metabolic health.

Indeed, recent studies have shown promise in improving sleep health, as Sirimon Reutrakul reported in “Sleep Interventions and Glucose Metabolism.” In a meta-analysis of sleep extension studies, behavioral change techniques (such as shaping knowledge, goals and planning, feedback and monitoring, and social support) were found to increase sleep duration by an average of 48 minutes per night [63]. After 2–6 weeks of sleep extension, some studies showed favorable changes in glucose metabolism indices, including lower fasting insulin, improved beta cell function, and better glycemic control [64–66], but negative findings were also reported [67]. Dr Reutrakul and colleagues investigated the use of a technology-assisted sleep intervention (“Sleep-Extend”) in a pilot study of women with previous history of gestational diabetes (a group at high risk of developing diabetes) and short sleep [68]. After 6 weeks, the Sleep-Extend group – who were given Fitbit fitness trackers, weekly digital content, and weekly coaching – slept ∼ 36 minutes longer and showed a favorable trend in fasting glucose levels and decreased fatigue [68]. These early data suggest possible glycemic benefits of behavioral sleep intervention technologies in people with diabetes, especially those at high risk, but they should be replicated in larger studies.

In “Stress and Sleep in Type 1 Diabetes,” Pamela Martyn-Nemeth presented the results of a technology-assisted sleep intervention study in individuals with type 1 diabetes. Type 1 diabetes requires constant self-management, often leading to diabetes distress or the feeling of being overwhelmed with the burden of managing this life-threatening condition. Fear of hypoglycemia is a major source of distress that impacts sleep, resulting in delayed sleep, waking to monitor blood glucose, or maintaining blood glucose levels higher to avoid hypoglycemia [69]. Poor sleep health is common among adults with type 1 diabetes, with 40% reporting short sleep duration (< 6–6.5 hours sleep per night) [70] and up to 51% reporting irregular sleep timing [71]. Just one night of restricted sleep is associated with greater insulin resistance in persons with type 1 diabetes [72]. To improve sleep and glycemic control in adults with type 1 diabetes, Dr Martyn-Nemeth and colleagues developed a technology-assisted behavioral sleep intervention (“Sleep-Opt-In”). For 8 weeks, individuals in the Sleep-Opt-In group had access to digital lessons, a sleep tracker, and weekly phone calls from a trained sleep coach. Afterward, they assessed the feasibility of Sleep-Opt-In and its effects on sleep duration, sleep regularity, glycemic parameters, and patient-reported outcomes [73]. The study results revealed that irregular sleep was a greater problem than insufficient sleep. The Sleep-Opt-In intervention led to improved sleep regularity, improved glucose parameters (CV% and time-in-range), reduced fatigue, and enhanced mood [73], suggesting that individuals with type 1 diabetes and irregular sleep may benefit from sleep optimization interventions.

### Environmental/Community Sources of Stress

Although genetic susceptibility, obesity, and sleep disturbances undoubtedly amplify diabetes risk, these factors fail to fully account for the global surge in diabetes rates. Recently, environmental contaminants acting as endocrine-disrupting chemicals (EDCs) have been implicated in the pathogenesis of diabetes. As Robert Sargis discussed in “Environmental Drivers of Diabetes Risk, Complications, and Disparities,” a variety of EDCs, from both natural and synthetic origin, can alter insulin secretion and action and disrupt glucose homeostasis [74–77]. They can even prime future generations for metabolic disease risk through epigenetic mechanisms [78]. Diabetogenic EDCs are generated by industrial activity, used in food production, and embedded into personal and home care products [77]. Low income and communities of color are disproportionately exposed to diabetogenic EDCs. Indeed, Dr Sargis and colleagues demonstrated that a Mexican-American population at high risk of developing diabetes had elevations in urinary EDCs (i.e., metals like arsenic, molybdenum, and copper), which was associated with reduced pancreatic beta cell function and insulin resistance [79]. Some EDCs used in plastic production, like phthalates and bisphenol A, are even incorporated into medications, medical equipment, and medical products [80]. Little has been done to understand and eliminate EDC exposures in medical care [80] and the environment in general [81]. To bridge the gaps between knowledge and action, Dr Sargis recommended the following courses of action: (1) screening and regulating chemicals based on their metabolism-disrupting effects; (2) understanding the impact of chemical mixtures on metabolic health; (3) evaluating the combined impacts of EDCs and traditional metabolic disease risk factors or underlying illnesses; (4) evaluating the toll of EDCs in the context of other social/structural determinants of health; (5) identifying and targeting sensitive windows of development for EDC-induced diabetes; (6) understanding how EDCs interact with climate change to threaten metabolic health; (7) evaluating interventions to reduce EDC levels and/or impact; and (8) testing and implementing public policies that reduce EDC exposures [82–85]. This comprehensive approach to identify and eliminate EDCs may help mitigate diabetes risk.

The COVID-19 pandemic has spurred increases in both diabetes incidence and cardiovascular disease [86,87]. Chronic stress promotes systemic inflammation and oxidative stress, which in turn, lead to endothelial dysfunction and atherosclerosis as well as insulin resistance and diabetes. In her lecture “Stress, Cardiovascular Disease, and Diabetes in the COVID-19 Era,” Amisha Wallia discussed how stress has contributed to these worsening health outcomes. A number of unique stressors coincided during the COVID-19 era, including workplace stressors (e.g., increased workload, poor work-life balance, job insecurity), community stressors (e.g., civil unrest, social isolation, resource shortages), and notably, healthcare system stressors (e.g., workforce strain, scarce medical resources). Even before the start of the COVID-19 pandemic, prediabetes and diabetes rates were increasing [88], while the diabetes workforce in the United States remained stagnant [89]. Meanwhile, a surplus of diabetes care and education specialists were being underutilized [90]. Interestingly, there is some evidence that glycemic control improved during pandemic-related lockdowns [91]. However, this was not always the case, especially for patients lacking access to diabetes management technology, such as telemedicine and continuous glucose monitoring [92]. These findings highlight stark differences across different populations based upon not just physiological factors but also social factors [93,94]. Future work must seek to understand these social contexts and their effects on cardiovascular disease and diabetes risk. Additionally, the COVID-19 era has emphasized the need to improve operation capacities for increased burden of care. Novel technologies, such as continuous glucose monitoring and telemedicine, may help optimize diabetes care pathways moving forward [95].

### Interventions to Mitigate the Effects of Stress on Diabetes

Diabetes has a disproportionate effect on racial/ethnic minorities and lower socioeconomic groups in terms of health outcomes and quality of care. In the U.S., the overall prevalence of diabetes (diagnosed and undiagnosed) in the U.S. is at 14.7%; however, the rates are higher at 17.4% for Blacks, 16.7% for Asians, and 15.5% for Hispanics compared to 13.6% for Whites [96]. Arleen Tuchman provided “Historical Reflections on Stress, Race, and Diabetes,” in which she highlighted the importance of considering the unique stressors experienced by different racial groups – not simply their genetics. By focusing on genes alone, we ignore stress and the physiological changes it elicits in our health. Paying attention to these stressors opens the door to exploring the role of racism and poverty as a root cause of negative health outcomes. This new knowledge will help inform the future of diabetes interventions.

Diabetes technologies provide many benefits, both metabolic (e.g., improved HbA1c) and psychological (e.g., decreased diabetes distress and fear of hypoglycemia). However, many disparities exist in technology adoption/uptake and long-term continued usage. These disparities in diabetes technology use remain, even in countries that fully fund technology use or when controlling for typical markers of socioeconomic status. What remains are clear disparities based on race and ethnicity. In “The Intersection Between Disparities in Technology Access, Uptake, and Continued Use and Psychosocial Stress,” Jill Weissberg-Benchell examined the challenges of implementing diabetes technology and how technology could be used to alleviate diabetes distress. Social determinants of health significantly impact diabetes distress, with higher levels of diabetes distress seen among youth and adults who identify as being from racial and ethnic minority backgrounds [97–99]. To reduce levels of emotional distress and depression in teens with type 1 diabetes, Dr Weissberg-Benchell and colleagues developed the randomized controlled trial STePS (supporting teen problem-solving) [100]. STePS is a resilience-focused intervention that teaches cognitive-behavioral and social problem-solving to reduce diabetes distress in teenagers [100]. After 3 years, participants in the intervention group reported significantly lower levels of diabetes distress [100]. Notably, the study enrollment was diverse, with 35% of participants identifying as racial or ethnic minorities [100]. Most studies are less diverse, and therefore, scientists and physicians should be cognizant that not all studies can be generalized to the entire population.

Screening and early diagnosis are critical to slow diabetes progression and prevent complications and early mortality [101]. However, disparities by race/ethnicity and socioeconomics exist in type 2 diabetes prevention, screening, and treatment [102]. Minority and underserved populations are frequently served by emergency departments of urban medical institutions. In the past, emergency departments have successfully implemented screening programs for HIV [103] and substance abuse [104]. But, for diabetes, there have been only a few short-term studies [105,106]. To address this gap, Kirstie Danielson and colleagues developed an emergency department diabetes screening program that was incorporated into daily clinical care [107]. Dr Danielson presented the results of this study in “A Novel Electronic Medical Record Diabetes Screening Program in an Urban Academic Hospital Emergency Department.” In this study, at-risk patients (≥ 45 years or 18–44 years with BMI ≥ 25) without a history of diabetes were flagged by the system so that a provider could add an HbA1c test to an ordered blood specimen. Of 8441 emergency department patients, 2576 patients were flagged by the system and 1085 patients had an abnormal HbA1c. Of those, 70% were consistent with prediabetes (HbA1c 5.7%–6.4%) and 30% diabetes (HbA1c ≥ 6.5%). Their novel screening in the emergency department identified a high number of patients with undiagnosed diabetes, particularly minority and poor patients. These results could inform future development of neighborhood and health care system interventions and policies to improve community diabetes outcomes. In addition, this approach could be adopted by other urban and rural emergency departments across the country. Future research will test the linkage of these new patients to diabetes education and care.

## Conclusion

At the conclusion of the Chicago Biomedical Consortium’s 19^th^ annual symposium, it was clear to all participants that stress plays a multifaceted role in diabetes, to the detriment of those living with the disease. Cellular, physiological, and community sources of stress all contribute to worsening diabetes outcomes. At the cellular level, pro-inflammatory and ER stress pathways in pancreatic beta cells and their surrounding microenvironment have been shown to promote beta cell dysfunction and death. However, because of their ubiquitous nature, future studies must address whether these pathways are able to be therapeutically targeted without significant off-target effects. As for physiological stress, obesity is the leading risk factor for type 2 diabetes. Obesity also makes diabetes much more difficult to manage and treat. Therefore, the goal is to find therapies that are effective in treating diabetes but also easily tolerated. Intermittent fasting has shown promise, but more studies will be needed to sort out the variable results obtained with different study designs. Another approach could be to target obesity at its source. While brain estrogen signaling appears to protect against obesity, the feasibility of targeting these pathways will need to be further investigated. Physiological stressors in diabetes – such as obesity and sleep disturbances – may respond particularly well to cognitive behavioral therapy, health technology (fitness and sleep trackers), and education and coaching. Within the environment and community, critical issues to address include patient exposure to diabetogenic chemicals in medicines and medical supplies, as well as the national shortage of diabetes healthcare providers.

Many presenters emphasized that racial/ethnic and socioeconomic disparities not only increase levels of stress and diabetes but also prevent access to diabetes care. Future research must seek to improve access to healthcare, for example, by expanding diabetes screening and tailoring intervention programs to meet patients’ needs. Still, there is much to be done to increase access to and adoption of diabetes technologies, like continuous glucose monitors, which could have a substantial impact on diabetes outcomes.

By alleviating diabetes-related stress at multiple levels – attacking the root causes of cellular stress and pancreatic beta cell death, reversing obesity and sleep loss, removing diabetogenic chemicals from our environment, addressing systemic racism, and improving our healthcare capabilities – we have the best chance of improving diabetes outcomes in the future.
